# Melatonin Treatment Alleviates Chilling Injury of Loquat Fruit via Modulating ROS Metabolism

**DOI:** 10.3390/foods13193050

**Published:** 2024-09-25

**Authors:** Jiahui Wan, Yanting Wu, Zhihong Tong, Wenbing Su, Hetong Lin, Zhongqi Fan

**Affiliations:** 1 Institute of Postharvest Technology of Agricultural Products, College of Food Science, Fujian Agriculture and Forestry University, Fuzhou 350002, China; 2 Key Laboratory of Postharvest Biology of Subtropical Special Agricultural Products, Fujian Province University, Fuzhou 350002, China; 3 Fruit Research Institute, Fujian Academy of Agricultural Science, Fuzhou 350013, China

**Keywords:** loquat, chilling injury, melatonin, ROS metabolism, antioxidant enzyme

## Abstract

Cold storage is one of the most effective methods to maintain postharvest fruit quality. However, loquat fruits are prone to chilling injury (CI) during cold storage, appearing as symptoms such as browning and pitting, which leads to quality deterioration and economic losses. In this study, the effects of melatonin on CI alleviation and the potential role of reactive oxygen species (ROS) metabolism in loquat fruit were investigated. The results showed that 50 μM melatonin was the optimal concentration to inhibit the increase in CI index and cell membrane permeability. Moreover, compared to control fruits, 50 μM melatonin inhibited the malonaldehyde (MDA) content, O_2_^−.^ production rate and H_2_O_2_ content (ROS accumulation) by 17.8%, 7.2% and 11.8%, respectively, during cold storage. Compared to non-treated loquats, 50 μM melatonin maintained higher levels of 1-diphenyl-2-picrylhydrazyl radical-scavenging ability and reducing power, as well as the contents of ascorbic acid (AsA) and glutathione (GSH). Additionally, 50 μM melatonin enhanced the activities of antioxidant enzymes, such as superoxide dismutase (SOD), catalase (CAT) and ascorbate peroxidase (APX) by increasing relevant gene expressions. The activities of SOD, CAT and APX were increased by up to 1.1-, 1.1- and 1.1-times (16 d) by melatonin, as compared with the control fruits. These findings indicate that melatonin mitigation of CI is involved in maintaining cellular redox apphomeostasis in loquat fruit during cold storage.

## 1. Introduction

Loquat, a member of the *Eriobotrya genus* in the *Rosaceae* family, is an evergreen small tree native to the warmer climatic regions of southern China. Currently, it is cultivated in over 30 countries worldwide [1]. The development of the loquat industry has effectively contributed to increasing farmers’ income and promoting rural economic growth. Loquat fruits are known for their traditional medicinal properties, such as helping to relieve coughs and moisturize the lungs, and the fruits are juicy, sweet and nutrient-rich, making them popular among consumers [2,3]. However, due to the perishability of loquat fruits and the fact that they are mostly harvested during the hot and rainy seasons, they are susceptible to microbial infestation, mechanical damage, high temperature and humidity, resulting in a short shelf life and difficulty maintaining fruit quality after harvest. Low-temperature storage and transportation are crucial for reducing postharvest spoilage, preserving fruit quality and delaying senescence [4,5]. Nevertheless, improper low-temperature treatment can cause chilling injury (CI) symptoms in loquat fruits, leading to browning, ion leakage and lignin accumulation, ultimately rendering the fruits commercially unviable and causing economic losses [6]. Therefore, investigating the mechanism of CI occurrence in loquat fruits and developing CI control techniques are significant for fruit storage, transportation and marketing.

When plants are exposed to adverse environmental conditions, such as cold, drought and salt stress, they respond by rapidly synthesizing reactive oxygen species (ROS), including superoxide radical anion (O_2_^−.^), hydrogen peroxide (H_2_O_2_) and hydroxyl radical (OH^−^) [7,8]. A controlled elevation in the intracellular ROS levels serves to stimulate the plant’s endogenous antioxidant defense mechanisms and hormonal signaling networks. This activation subsequently induces the expression of downstream stress-response genes, initiating the plant’s adaptive reactions to external stressors [9,10]. Nevertheless, the excessive accumulation of ROS can transform them into deleterious metabolites, which engage in peroxidation reactions with phospholipids and membrane receptor proteins, producing malonaldehyde (MDA) and causing cellular structural damage, ultimately leading to senescence and death of plant cells [11]. A recent study has revealed that the contents of H_2_O_2_ and MDA were induced by cold, and the activities of antioxidant enzymes, including superoxide dismutase (SOD), catalase (CAT), ascorbate peroxidase (APX), were slightly increased in peach fruit during cold storage, and the treatment of trehalose could improve antioxidant enzyme activities and CI tolerance [12]. Also, cold-shock treatment could mitigate CI in peach fruit by promoting the activities of SOD, CAT and APX, which coincided with the lower levels of ROS content [13]. In general, cold storage triggers oxidative damage due to the increased ROS production and eventually leads to CI symptoms; however, antioxidative enzyme activity plays a very imperative role in ROS-scavenging to alleviate oxidative stress [14,15].

Melatonin, an indolamine compound widely found in plants, has been considered a natural, highly effective and safe preservative for fruits and vegetables. It plays a crucial role in regulating their ripening and senescence, scavenging reactive oxygen species free radicals and enhancing resistance to both biotic and abiotic stresses. For instance, the application of melatonin promoted endogenous melatonin and hydrogen sulfide accumulation by triggering the relative gene expression and reduced the accumulation of H_2_O_2_ and MDA, resulting in senescence delay and browning symptoms alleviation in sweet cherry fruit during cold storage [16]. In zucchini fruit, melatonin alleviated CI, suppressed mass loss, improved proline and γ-amminobutyric acid accumulation and reduced oxidative stress [17]. Similarly, melatonin mitigated CI development in bananas by promoting phospholipids and unsaturated fatty acid accumulation, decreasing the production of reactive oxygen species (ROS) and the gene expression of polyphenol oxidase (PPO) [18]. In peach fruit, melatonin improved CI tolerance by reducing the H_2_O_2_ content but increasing the expressions of antioxidant enzyme genes and the contents of ascorbic acid (AsA) [19]. Collectively, these studies imply that melatonin has positive effects in alleviating the CI of fresh products and, more importantly, the enhancement of chilling tolerance, which is induced by melatonin in association with improved antioxidant ability. Here, our research focuses on melatonin’s role in reducing the chilling injury of postharvest loquat fruit. We investigate the optimal melatonin concentration and its impact on ROS and related physiological and molecular indicators during cold storage. Our work delves into melatonin’s mode of action in modulating ROS homeostasis under cold stress, providing insights into improving postharvest fruit quality. We propose melatonin as a natural and sustainable alternative to traditional chemical treatments, aligning with eco-friendly agricultural practices. 

## 2. Materials and Methods

### 2.1. Fruit Material and Treatments

On 27 April 2024, Loquat fruits (*Eriobotrya japonica* L. cv. Guifei) at 80–90% maturation (the commercial maturity, 142 days after full flowering) were taken from an orchard in Putian, Fujian, Southeast China. Intact fruits of uniform size and color were selected for the experiment. In the preliminary experiment, loquat fruits were soaked in different concentrations of melatonin (25 μM, 50 μM, 75 μM, 100 μM) and distilled water (control group) for 10 min, respectively. After air-drying, they were packaged and stored at a relative humidity of 90% and (4 ± 1) °C for 20 days. The most effective concentration of melatonin to inhibit CI and cell membrane permeability was used in the formal experiment. The pulp tissues of loquats were sampled at 0, 4, 8, 12, 16 and 20 d, and stored at −80 °C for further use (a total of 600 fruits) [20,21]. 

### 2.2. Measurement of CI Index, Cell Membrane Permeability and MDA Content

The CI index was visually evaluated according to a previous method [22]. The CI index was scored based on the extent of browning on the fruit surface: 0, no visible CI symptom; 1, slight CI symptom (<5%); 2, moderate CI symptom (5–25%); 3, severe CI symptom (25–50%); 4, very severe CI symptom (>50%), and the result was calculated as Σ (CI level × the ratio of corresponding fruit in each CI level) [23]. A total of 120 fruits were used in CI index analysis, among them, eight fruits were considered as one group, three groups (24 fruits) were considered as three biology replicates, and the 120 fruits were used in five treatments, including control, 25 μM-, 50 μM-, 75 μM- and 100 μM-melatonin treatment. The detailed data of the CI levels on each sampling day are listed as a Supplementary File in Table S1.

Cell membrane permeability was measured following the method of Xie et al. [24]. Procedure: (a) Loquat discs (2 g) with a diameter of 0.5 cm were taken from 10 fruits. (b) Immersed in 25 mL distilled water, the initial conductivity (*L_0_*) was determined by a desktop conductivity meter. (c) After 3 h, the solution was boiled for 30 min and cooled, and the final conductivity (*L_1_*) was measured. (d) The result was calculated as *L_0_*/*L_1_* × 100, and expressed as % [25]. A total of 300 fruits were used in the cell membrane permeability analysis, among them, loquat discs (2 g) with a diameter of 0.5 cm were taken from 10 fruits. This was repeated three times, and each of the 10 fruits was taken on sampling days 0, 4, 8, 12, 16, and 20 (a total of 60 fruits), as well as five treatments, including the control, 25 μM-, 50 μM-, 75 μM- and 100 μM-melatonin treatment.

Loquat tissues (2 g) from 10 fruits were taken to perform the MDA content determination; this was measured following the thiobarbituric acid colorimetric method. Procedure: (a) Loquat tissues (2 g) were extracted by 10 mL 10% trichloroacetic acid (TCA) solution and centrifuged. (b) A total of 4 mL extraction solution was mixed with 4 mL 0.67% thiobarbituric acid (TBA) and reacted in a boiling water bath for 20 min. (c) The absorbance value was determined at 450 nm, 532 nm and 600 nm; the result was expressed as μmol kg^−1^ FW (fresh weight) [26].

### 2.3. Quantitative of O_2_^−.^ Production Rate and H_2_O_2_ Content

The quantifications of the O_2_^−.^ production rate and H_2_O_2_ content were performed following the description of Cao et al. [27] and Li et al. [28]. 

The following was applied for O_2_^−.^ production rate determination: (a) Loquat tissues (2 g) were extracted by 10 mL phosphate-buffered saline (PBS) (pH 7.8, 50 mM, containing 1 mM ethylene diamine tetraacetic acid (EDTA)) and centrifuged. (b) A total of 1 mL of the obtained extraction solution was mixed with 1 mL PBS buffer (pH 7.8, 50 mM) and 1 mL 1 mM hydroxylamine hydrochloride in sequence. (c) This was placed in a constant temperature water bath at 25 °C for 1 h. The reaction solution without insulation for 1 h was used as the control. (d) Add 1 mL 17 mM p-aminobenzenesulfonic acid solution and 1 mL 7 mM a-naphthylamine solution for 20 min at 25 °C. (e) Immediately measure the OD value at 530 nm. (f) The results are expressed as mmol kg^−1^ min^−1^ FW based on a sodium nitrite standard curve. 

For H_2_O_2_ content determination, (a) loquat tissues were extracted by acetone and then centrifuged. (b) A total of 1 mL of the obtained extracting solution was mixed with 100 µL 10% titanium tetrachloride (TiCl_4_) solution and 200 µL concentrated ammonia water (NH_3_·H_2_O), reacted for 5 min, and centrifuged. (c) Discard the supernatant and wash the precipitate three times with acetone; then, dissolve the precipitate in 3 mL 1 M H_2_SO_4_. (d) The absorbance value was determined at 412 nm and the results are expressed as mol kg^−1^ FW.

### 2.4. Determination of AsA and GSH Contents

The determination of the AsA and glutathione (GSH) contents was based on the method of Chumyam et al. [29]. 

For the AsA content determination, (a) loquat tissues (2 g) were extracted by 5% TCA and centrifuged. (b) The 3 mL extraction solution was mixed with 3 mL 5% TCA and 3 mL anhydrous ethanol. (c) Add 1.5 mL 0.4% H_3_PO_4_-ethanol, 3 mL 0.5% phenanthroline ethanol, and 1.5 mL 0.03% FeCl_3_-ethanol in sequence. (d) React at 30 °C for 90 min. (e) The absorbance of the reaction solution was measured at 534 nm and the result is expressed as g kg^−1^ FW. 

For the GSH content measurement, (a) loquat tissues (2 g) were extracted by 5% TCA (containing 5mM EDTA-Na_2_) and centrifuged. (b) The 1 mL extraction solution was mixed with 1 mL of PBS buffer (pH 7.7, 0.1 M) and 0.5 mL of 4 mM dithionitrobenzoic acid (DTNB) solution into one tube. (c) The 1 mL extraction solution was mixed with 1 mL PBS buffer (pH 7.7, 0.1 M) and 0.5 mL PBS buffer (pH 6.8, 0.1 M) in the other tube. (d) Keep both tubes at 25 °C for 10 min. (e) The absorbance of the reaction solution was measured at 412 nm and the result is expressed as g kg^−1^ FW.

### 2.5. Analyses of Free Radical-Scavenging Ability and Reducing Power 

The analyses of reducing power and free radical-scavenging ability followed the method of Duan et al. [30]. 

The extracting solution: loquat tissues (2 g) were extracted by 10 mL 80% ethanol and gathered after centrifugation. 

For the reducing power analysis, (a) 0.2 mL extracting solution was mixed with 2.5 mL PBS (pH 6.6, 0.2 M), 2.5 mL 1% potassium ferricyanide, and 2.5 mL 10% TCA. (b) Centrifuge for 20 min. (c) Take the same volume of supernatant and distilled water and mix them with 1 mL of 0.1% ferric chloride. (d) The absorbance of supernatant was measured at 700 nm and the result is expressed as g kg^−1^ FW. 

For the free radical-scavenging ability analysis, (a) 1.5 mL extracting solution was mixed with 1.5 mL DPPH (60 µM)—ethanol solution. (b) This was held in a dark place at room temperature. (c) The absorbance of the mixture solution was measured at 517 nm and the result is expressed as a %. 

### 2.6. Estimations of SOD, CAT and APX Activities 

The estimations of SOD, CAT and APX activities were based on the description of Li et al. [31]. 

For the extracting solution, loquat tissues (2 g) were extracted using 10 mL of PBS buffer (pH 7.0, 50 mM), and gathered following centrifugation. 

For the SOD activity determination, (a) 0.1 mL extracting solution was mixed with 3 mL SOD reaction medium (containing 13 mM nitrotetrazolium blue chloride (NBT), PBS buffer (pH 7.8, 50 mM), 1.5 µM riboflavin and 63 µM methionine). (b) The reaction was illuminated for 30 min under 4000 Lux, and the reaction was immediately terminated in the dark. (c) The absorbance of the mixture solution was detected at 560 nm and the result is expressed as 10^6^ U kg^−1^ protein. (d) One unit of SOD activity was defined as the inhibits 50% NBT photochemical reduction per minute. 

For CAT activity determination, (a) 0.2 mL extracting solution was mixed with 2.8 mL of 20 mM H_2_O_2_ for 10 min at 30 °C. (b) The reaction was terminated by H_2_SO_4_, the absorbance of the mixture solution was detected at 240 nm and the result is expressed as 10^6^ U kg^−1^ protein. (c) One unit of CAT activity was defined as the enzymatic amount causing the absorbance changes at 0.01 per minute. 

For the APX activity determination, (a) 0.2 mL extracting solution was mixed with PBS buffer (pH 7.7, 50 mM, containing 0.5 mM AsA) and 0.5 mL 2 mM H_2_O_2_ for 20 min at 30 °C. (b) The mixture was terminated by 2 mL of 20% TCA. (c) The absorbance was detected at 290 nm and the result is expressed as 10^6^ U kg^−1^ protein. (d) One unit of APX activity was defined as the enzymatic amount causing the absorbance changes at 0.01 per minute. 

For protein content determination, 0.1 mL extracting solution was mixed with 10 mL Coomassie brilliant blue G-250. The absorbance of the mixture solution was measured at 595 nm after 2 min. A bovine serum albumin standard solution was used as the standard curve.

### 2.7. RNA Extraction and RT-qPCR Analysis

Total RNA was extracted from loquat pulp using a plant RNA isolation kit (Tiangen, Beijing, China). cDNA was synthesized using the First-strand cDNA synthesis mix kit (Lablead, Beijing, China). RT-qPCR was conducted using the SYBR qPCR mix kit (Vazyme, Nanjing, China) on a CFX96 Real-Time PCR System (Bio-Rad, Hercules, CA, USA), and *EjACT* (GenBank no. JN004223) was used as the internal reference gene [32]. All primers used in this study are listed in Table S4.

### 2.8. Statistical Analysis

All experiments used at least three biology replicates and followed the principle of complete randomization. A significance differences analysis was analyzed by Student’s *t*-test on Statistical Product and Service Solutions (SPSS) statistical software (version 22.0) (* *p* < 0.05, ** *p* < 0.01).

## 3. Results

### 3.1. Effects of Melatonin on Fruit Appearance, CI Index and Cell Membrane Permeability 

**Figure 1:**
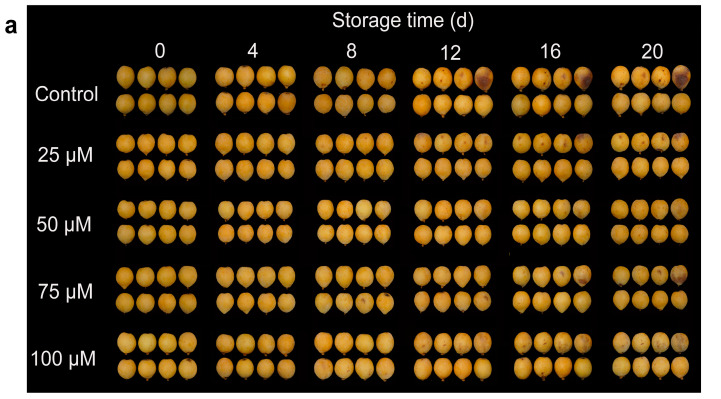

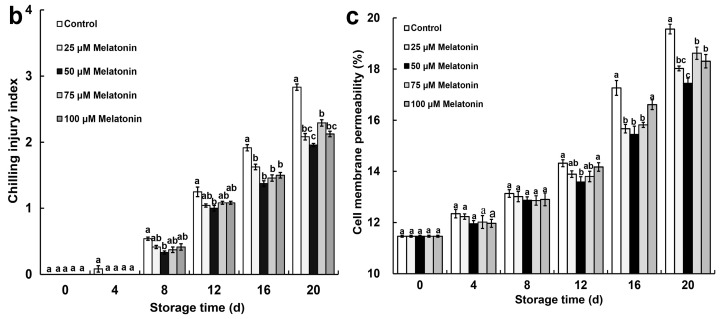
Melatonin treatment alleviates the development of CI. (**a**) Appearance, (**b**) CI index and (**c**) cell membrane permeability of loquat fruits stored at 4 °C for 20 days. Data are mean ± SD of three biology replicates. Different letters represent significant differences at *p* < 0.05.

CI symptoms were evaluated in loquat fruits during cold storage. As shown in Figure 1a, non-treated loquat fruits that were held in a cold environment for 20 days displayed serious CI symptoms, but the melatonin treatment alleviated CI development in fruits (Figure 1a). The CI index increased continuously throughout the whole storge, but melatonin effectively retarded the rising trend. The concentration of 50 μM was found to be the most effective in suppressing the CI index increase in the loquats. The concentrations that were higher (75 μM and 100 μM) or lower (25 μM) than 50 μM did not have any additional beneficial effect (Figure 1b). Cell membrane permeability exhibited an increasing trend in non-treated and melatonin-treated loquat fruits (Figure 1c). Overall, the increase in cell membrane permeability of the control group maintained higher levels compared to the melatonin-treated fruits. After 20 days of storage, the cell membrane permeability in the 50 μM melatonin-treated fruits was 17.4%, which was 10.8% lower than those of the non-treated loquats.

### 3.2. Effects of Melatonin on MDA Content, O_2_^−.^ Production Rate and H_2_O_2_ Content

**Figure 2:**
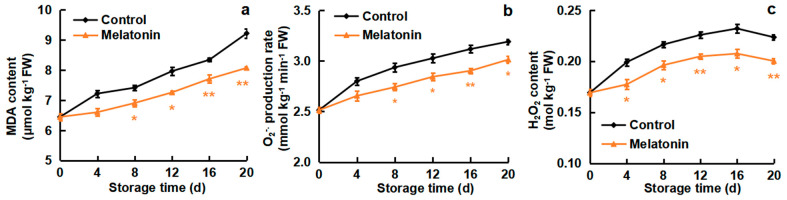
Effect of melatonin treatment on (**a**) MDA content, (**b**) O_2_^−.^ production rate and (**c**) H_2_O_2_ content of loquat fruits stored at 4 °C for 20 days. Data are mean ± SD of three biology replicates. * and ** represent the significant differences at *p* < 0.01 or *p* < 0.05 between the control and melatonin-treated fruits on the same storage day, respectively.

The best concentration of melatonin (50 µM) was used for further investigation. The changes of MDA content, O_2_^−.^ production rate and H_2_O_2_ content are shown in Figure 2. MDA content in all cold-stored loquat fruits gradually increased with the extension of storage time. Significant differences were found on storage days 8, 12, 16 and 20. MDA content of non-treated and melatonin-treated fruits increased from 6.4 μmol kg^−1^ on day 0 (the harvest day) to 9.2 μmol kg^−1^ and 8.1 μmol kg^−1^ on day 20 (the last storage day) (Figure 2a). As displayed in Figure 2b, cold storage induced the increase in the O_2_^−.^ production rate, but melatonin treatment retarded the increase. The maximum value of O_2_^−.^ production rate in control fruits was obtained by day 20, which showed a 1.3-fold increase in comparison with day 0, respectively. Correspondingly, and O_2_^−.^ production rate was increased by 1.2-fold in melatonin-treated fruits at day 20 compared to day 0. For H_2_O_2_ content, melatonin treatment effectively inhibited the increase in the whole storage time (Figure 2c). On storage day 16, H_2_O_2_ content in control and melatonin-treated loquats elevated by 37.1% and 22.6%, compared with day 0, respectively. Then, a slight decrease in H_2_O_2_ content was observed in both groups within day 16–20. These results demonstrated that melatonin treatment could reduce the accumulation of MDA, O_2_^−.^ and H_2_O_2_ content in loquat fruits during cold storage. 

### 3.3. Effects of Melatonin on AsA and GSH Contents

**Figure 3:**
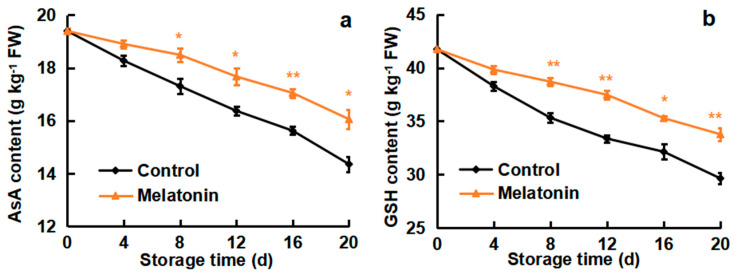
Effect of melatonin treatment on (**a**) AsA content and (**b**) GSH content of loquat fruits stored at 4 °C for 20 days. Data are mean ± SD of three biology replicates. * and ** represent the significant differences at *p* < 0.01 or *p* < 0.05 between the control and melatonin-treated fruits on the same storage day, respectively.

The contents of AsA and GSH exhibited a continuous decreasing trend in loquat fruits during cold storage in both control and melatonin treatment. As shown in Figure 3, the decrease in AsA and GSH contents was lower in the non-treated loquats as compared to the melatonin-treated fruits. Melatonin inhibited the decrease in AsA and GSH contents. After 20 days of cold storage, the AsA and GSH contents in melatonin fruits were 16.1 and 33.8 g kg^−1^, while, in the control loquats, they were 14.4 and 29.6 g kg^−1^. The loquats treated with melatonin showed higher AsA content (1.1-fold) and GSH content (1.1-fold) in contrast with the non-treated fruits. These findings revealed that melatonin treatment could delay the reduction in the AsA and GSH contents during cold storage.

### 3.4. Effects of Melatonin on DPPH Radical-Scavenging Ability and Reducing Power

**Figure 4:**
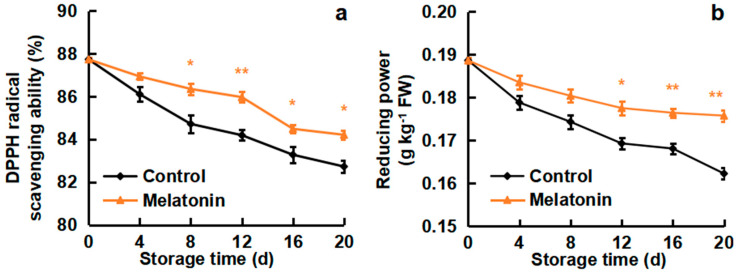
Effect of melatonin treatment on (**a**) DPPH radical-scavenging ability and (**b**) reducing power of loquat fruits stored at 4 °C for 20 days. Data are mean ± SD of three biology replicates. * and ** represent the significant differences at *p* < 0.01 or *p* < 0.05 between the control and melatonin-treated fruits on the same storage day, respectively.

DPPH radical-scavenging ability and reducing power showed a similar downtrend in loquat fruits during cold storage in both control and melatonin treatment (Figure 4). Melatonin treatment significantly delayed the decrease in the DPPH radical-scavenging ability and reducing power. During the storage, DPPH radical-scavenging ability was reduced by 5.7% in the control fruits, while it only decreased by 4.0% in the melatonin-treated fruits (Figure 4a). Similarly, the reducing power following melatonin treatment was 8.3% higher than the non-treated loquats on the last storage day (day 20) (Figure 4b). These results suggest that melatonin treatment resulted in a greater ability to scavenge ROS in the cold-stored loquat fruits. 

### 3.5. Effects of Melatonin on the Activities of SOD, CAT and APX

**Figure 5:**
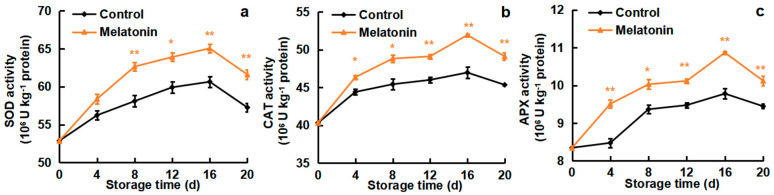
Effect of melatonin treatment on (**a**) SOD activity, (**b**) CAT activity and (**c**) APX activity of loquat fruits stored at 4 °C for 20 days. Data are mean ± SD of three biology replicates. * and ** represent the significant differences at *p* < 0.01 or *p* < 0.05 between the control and melatonin-treated fruits on the same storage day, respectively.

As shown in Figure 5a, the SOD activity in the cold-stored loquats displayed an upward tendency from day 0 to 16, followed by a decline during the last four days of storage. Compared with the control loquats, the SOD activity of the melatonin treatment loquats was significantly (*p* < 0.05) higher in the whole storage period. Figure 5b shows that the CAT activity in the treated and non-treated fruits increased and peaked on day 16, with 49.1 and 45.4 10^6^ U kg^−1^ of protein, respectively, then declined from day 16 to 20. CAT activity changes in the melatonin group were maintained higher than those in the control group. Figure 5c displays that the APX activity in the melatonin group increased gradually from day 0 to day 16, changing from 8.3 to 10.9 10^6^ U kg^−1^ protein, while, in the control group, it changed from 8.3 to 9.8 10^6^ U kg^−1^ of protein. The activity of APX in the melatonin-treated fruits was higher than that in the control fruits during the whole cold storage period.

### 3.6. Effects of Melatonin on the Expressions of EjSODs, EjCATs and EjAPXs

**Figure 6:**
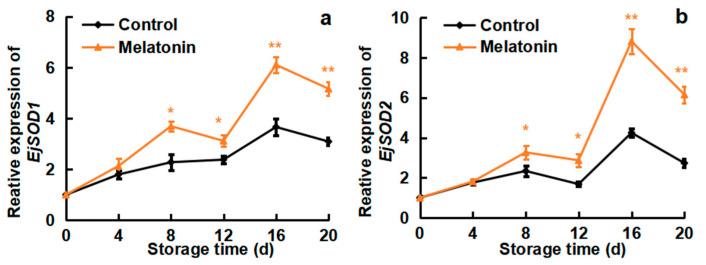

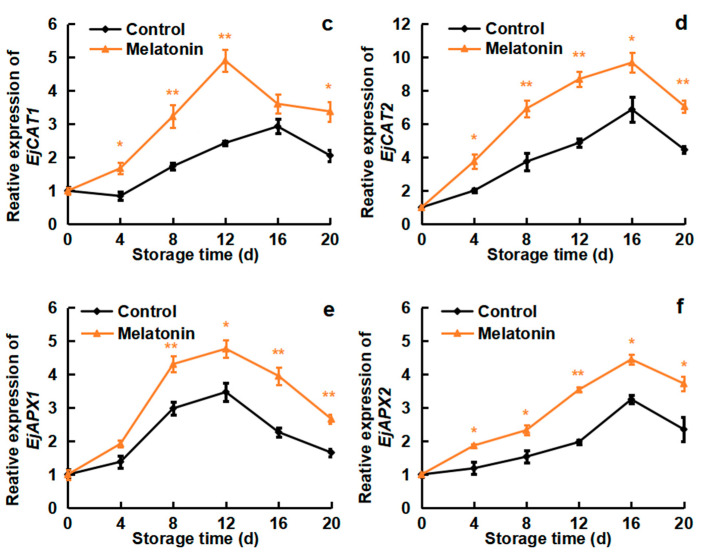
Effect of melatonin treatment on the expressions of the antioxidant enzyme genes (**a**,**b**) EjSOD1 and EjSOD2, (**c**,**d**) EjCAT1 and EjCAT2, and (**e**,**f**) EjAPX1 and EjAPX2 of loquat fruits stored at 4 °C for 20 days. Data are mean ± SD of three biology replicates. * and ** represent the significant differences at *p* < 0.01 or *p* < 0.05 between the control and melatonin-treated fruits on the same storage day, respectively.

To investigate the changes in antioxidant enzyme gene expression during cold storage, RT-qPCR was performed. As shown in Figure 6a, the expressions of *EjSOD1* and *EjSOD2* in the control and melatonin-treated fruits increased and peaked on day 16, which was in accordance with the increase in SOD activity. Compared to the control loquats, melatonin treatment maintained higher expressions of *EjSOD1* and *EjSOD2*. On day 16 of storage, *EjSOD1* and *EjSOD2* expressions in melatonin-treated loquats were 1.7- and 2.1-fold higher than in the control fruits, respectively. Additionally, the expressions of *EjCAT1/2* (Figure 6b) and *EjAPX1/2* (Figure 6c) in both the control and treated loquats showed an upward trend during the early storage period, then declined slightly. During the whole storage time, melatonin treatment displayed remarkably higher expressions of *EjCAT1/2* and *EjAPX1/2* than those in the control group. The *EjCAT1* and *EjCAT2* expressions in the melatonin-treated loquats were 64.1% and 58.3% higher on the 20th day compared with the control fruits, respectively (Figure 6b). The expressions of *EjAPX1* and *EjAPX2* in the melatonin-treated loquats were 60.8% and 58.3% higher on the 20th day compared with the control fruits, respectively (Figure 6c).

## 4. Discussion

Cold storage is an effective approach for preserving loquat fruits, efficiently controlling nutrient depletion, senescence and postharvest deterioration, thus extending the freshness duration and shelf life of loquats [33]. Nevertheless, Loquat fruit is susceptible to CI during cold storage, the distinct occurrence of CI induces appearance browning, lignin accumulation and fruit firmness, leading to quality reduction [34]. CI symptoms aggravate ROS over-accumulation and cell membrane peroxidation [35]. A previous study has shown that melatonin treatment could improve cold tolerance in several fruit species, including peach [19], Cherimoya [36], sapota [37] and banana [38]. These effects have been attributed to the antioxidant ability of cells and the integrality of the cell membrane. Here, CI symptoms were obviously reduced by melatonin treatment, especially with 50 μM doses (Figure 1a), and CI index (Figure 1b), cell membrane permeability (Figure 1c) and MDA content (Figure 2a) were likewise inhibited, suggesting that melatonin treatment effectively reduced the CI symptoms of loquat fruit. 

ROS have crucial roles in the plant response to abiotic stress; oxidative damage is considered to be an early response to cold environments. Low temperature stimulates ROS excessive accumulation, including O_2_^−.^ and H_2_O_2_ production, resulting in CI occurrence [39]. The AsA and GSH are important antioxidants that are able to substantially scavenge ROS, maintaining a cellular redox state [40,41]. In the present study, the O_2_^−.^ production rate and H_2_O_2_ content were induced by cold storage, but melatonin treatment efficiently delayed the ROS increase (Figure 2), leading to a lower level of membrane peroxidation in loquats. Moreover, melatonin-treated loquat fruits maintained higher levels of AsA and GSH contents as compared to the control group (Figure 3). Furthermore, melatonin treatment enhanced DPPH scavenging ability and reducing power, which are significant indicators affecting antioxidant capacity (Figure 4). Similar results were reported in the melatonin-improved chilling tolerance of peach fruit; melatonin treatment inhibited H_2_O_2_ accumulation and kept higher levels of AsA content [19]. Also, CaCl_2_ treatment alleviated the CI of loquats by reducing the production of ROS but increasing the DPPH scavenging ability and resulted in the fruits retaining higher contents of AsA and GSH [26]. γ-aminobutyric acid (GABA) treatment resulted in a suppression of the O_2_^−.^ production rate, maintenance of higher levels of AsA and GSH, and retention of a greater capacity for scavenging radicals [42]. These findings indicated that the effects of melatonin treatment on CI reduction were associated with ROS reduction, antioxidant substances accumulation and radical scavenging-ability enhancement. 

Antioxidant enzymes, such as SOD, CAT and APX, are widely involved in CI alleviation. SOD can transform the excessively produced O_2_^−.^ into H_2_O_2_, whereas CAT and APX facilitate the conversion of H_2_O_2_ into H_2_O and O_2_ [43]. Studies have shown that these antioxidant enzymes were associated with the inhibition of oxidative damage and the improvement of cold tolerance in banana [44], peach [19] and sapota fruit [37]. The activity and the expression of SOD were up-regulated by CaCl_2_ treatment, and the browning of pear fruit was inhibited during cold storage [45]. GABA-suppressed CI in Chinese olives was associated with improved SOD, CAT and APX activities [42]. Hot water treatment-alleviated CI in banana fruit was related to higher expressions of *MaAPXs* [44]. The activities of SOD and CAT, and the expression of *EjSOD* and *EjCAT*, were increased by CaCl_2_ treatment in loquat fruit, contributing to CI alleviation [26]. In agreement with these results, melatonin-treated loquats maintained higher activities of SOD, CAT and APX (Figure 5), as well as higher expression levels of *EjSODs*, *EjCATs and EjAPXs* (Figure 6), when compared with control fruit. It might thus be concluded that melatonin has certain effects on the activity of the antioxidant enzymes activity and antioxidant enzyme gene expressions in loquats subjected to cold and its final levels in the storage period.

**Figure 7:**
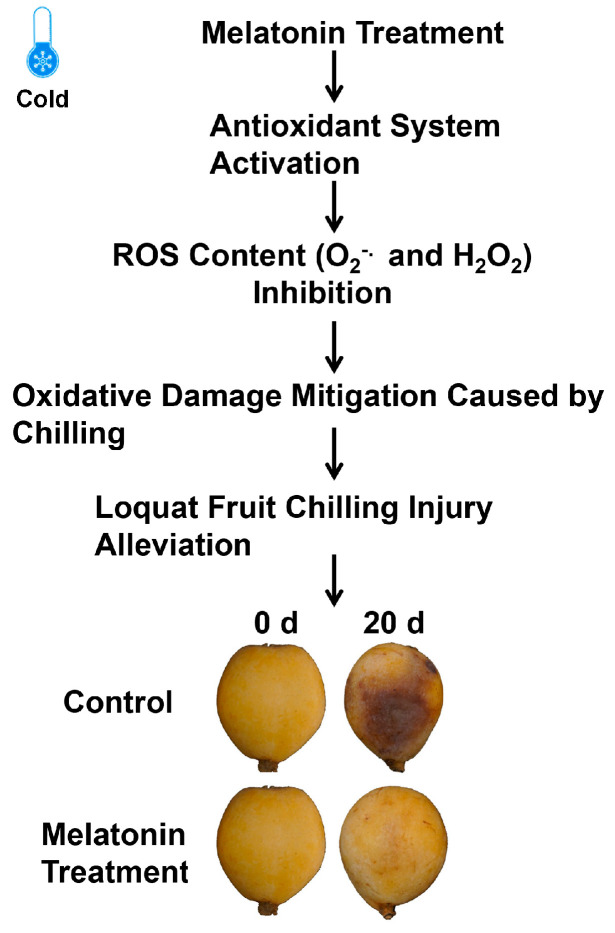
A working model of melatonin-alleviating CI in postharvest loquat fruit.

A working model for melatonin alleviating CI in postharvest loquat fruit was proposed based on these findings provided herein (Figure 7). The accumulation of O_2_^−.^ and H_2_O_2_ in cold-stored loquats could serve as signals to stimulate the antioxidant system and subsequently improve cold tolerance. The activated scavenging systems, including enzymatic and non-enzymatic systems, in turn, eliminated the overproduction of ROS, thereby suppressing the oxidative damage caused by cold in melatonin-treated loquats, which also contributed to the improvement of cold tolerance. Collectively, melatonin could regulate antioxidant enzymes and genes and also promote cellular antioxidants, therefore mitigating the development of CI. 

## 5. Conclusions 

In conclusion, the treatment of harvested loquat fruit with melatonin effectively alleviated CI symptoms caused by cold storage, which was evidenced by increased cell membrane permeability and MDA content and excessive production of O_2_^−.^ and H_2_O_2_ in loquat fruit during storage. Moreover, melatonin treatment improved the contents of endogenous antioxidant substances (AsA and GSH) and promoted the DPPH radical-scavenging ability of cells, as well as the activity and gene expression of antioxidant enzymes (SOD, CAT and APX). These results provide valuable insights for improving cold tolerance in loquat fruit through regulating ROS homeostasis by melatonin treatment. Nevertheless, additional molecular evidence is needed to elucidate how melatonin induces resistance to cold stress. 

## Data Availability

The original contributions presented in the study are included in the article/Supplementary Materials, further inquiries can be directed to the corresponding author.

## Supplementary Materials

The following supporting information can be downloaded at: https://www.mdpi.com/article/10.3390/foods13193050/s1, Table S1. The data for calculating chilling injury index. Table S2. The brand and model of all equipment used in this study. Table S3. The reagent and their density used in this study. Table S4. The List of primers used in this study.
