# Supplementary material for: Melatonin Treatment Alleviates Chilling Injury of Loquat Fruit via Modulating ROS Metabolism
Source: Foods. 2024 Sep 25;13(19):3050. doi: 10.3390/foods13193050 (PMC11476320; doi:10.3390/foods13193050)
Supplement: Supplementary file 1 [file foods-13-03050-s001.zip › foods-3209275-supplementary.pdf]

Supplementary Table S1. The data for calculating chilling injury index.

		Level 0	Level 1	Level 2	Level 3	Level 4	CI index
0 d	CK-1	8					0
	CK-2	8					0
	CK-3	8					0
	25 μM-1	8					0
	25 μM-2	8					0
	25 μM-3	8					0
	50 μM-1	8					0
	50 μM-2	8					0
	50 μM-3	8					0
	75 μM-1	8					0
	75 μM-2	8					0
	75 μM-3	8					0
	100 μM-1	8					0
	100 μM-2	8					0
	100 μM-3	8					0
4 d	CK-1	6	2				0.25
	CK-2	8					0
	CK-3	8					0
	25 μM-1	8					0
	25 μM-2	8					0
	25 μM-3	8					0
	50 μM-1	8					0
	50 μM-2	8					0
	50 μM-3	8					0
	75 μM-1	8					0
	75 μM-2	8					0
	75 μM-3	8					0
	100 μM-1	8					0
	100 μM-2	8					0
	100 μM-3	8					0
8 d	CK-1	4	4				0.5
	CK-2	4	4				0.5
	CK-3	3	5				0.63
	25 μM-1	4	4				0.5
	25 μM-2	5	3				0.38
	25 μM-3	5	3				0.38
	50 μM-1	5	3				0.38
	50 μM-2	6	2				0.25
	50 μM-3	5	3				0.38
	75 μM-1	4	4				0.50
	75 μM-2	5	3				0.38

	75 μM-3	6	3				0.25
	100 μM-1	4	4				0.50
	100 μM-2	4	4				0.50
	100 μM-3	6	2				0.25
12 d	CK-1		6	1		1	1.50
	CK-2		7	1			1.13
	CK-3		7	1			1.13
	25 μM-1		7	1			1.13
	25 μM-2		8				1.00
	25 μM-3		8				1.00
	50 μM-1	1	7				0.88
	50 μM-2		7	1			1.13
	50 μM-3		8				1.00
	75 μM-1		7	1			1.13
	75 μM-2		8				1.00
	75 μM-3		7	1			1.13
	100 μM-1		7	1			1.13
	100 μM-2		8				1.13
	100 μM-3		7	1			1.00
16 d	CK-1		3	3	1	1	2.00
	CK-2		3	3	1	1	2.00
	CK-3		3	4	1		1.75
	25 μM-1		3	4	1		1.75
	25 μM-2		4	4			1.50
	25 μM-3		3	5			1.63
	50 μM-1		5	2	1		1.50
	50 μM-2		5	3			1.38
	50 μM-3		6	2			1.25
	75 μM-1		4	3	1		1.63
	75 μM-2		5	3			1.38
	75 μM-3		5	3			1.38
	100 μM-1		4	3	1		1.63
	100 μM-2		5	3			1.38
	100 μM-3		5	2	1		1.50
20 d	CK-1			4	2	2	2.75
	CK-2			2	4	2	3.00
	CK-3			4	2	2	2.75
	25 μM-1		2	4	2		2.00
	25 μM-2		2	4	2		2.00
	25 μM-3			6	2		2.25
	50 μM-1		2	5	1		1.88
	50 μM-2		1	6	1		2.00
	50 μM-3		1	6	1		2.00

	75 μM-1		3	1	2	2	2.38
	75 μM-2		1	5	2		2.13
	75 μM-3		2	3	1	2	2.38
	100 μM-1		1	5	2		2.13
	100 μM-2		1	5	1	1	2.25
	100 μM-3		2	4	2		2.00

Supplementary Table S2. The brand and model of all equipment used in this study.

**Equipment**	**Model**	**Brand**
Electronic analytical balance	BSA224S	Sartorius
Microplate Reader	Spectra Max 190	Molecular Devices
Centrifuge	GL-20G-II	Shanghai Anting
Water bath	HH-4	Guo Hua
Real-Time PCR System	CFX Duet	Bio-Rad
Electromagnetic furnace	C21-RT2140	Midea
Conductivity meter	CON 700	Eutech

Supplementary Table S3. The reagent and their density used in this study.

**Reagent**	**Density**
Trichloroacetic acid (TCA)	5% (w/v), 10% (w/v)
Thiobarbituric acid (TBA)	0.67% (w/v)
Ethylenediaminetetraacetic acid disodium salt (EDTA-Na_2)	5 mM
phosphate buffered saline（PBS）	50 mM pH 7.8<br>50 mM pH 7.0<br>50 mM pH 7.7<br>0.1 M pH 7.7<br>0.1 M pH 6.8<br>0.2 mM pH 6.6
L-Ascorbic acid (ASA)	

Hydroxylamine hydrochloride ($NH_2OH \cdot HCL$)	1 mM
Sulfanilic acid	17 mM
α-Naphthylamine	7 mM
Nitro-blue tetrazolium (NBT)	63 μM
Methionine	13 mM
Ethanol absolute	80% (v/v)
Concentrated sulfuric acid	1 M, 10 % (v/v)
Phosphoric acid	
4,7-Diphenyl-1,10-phenanthroline	0.5% (w/v)
Ferric chloride	0.1% (w/v), 0.03% (w/v)
5,5'-Dithiobis	4 mM
Potassium ferrocyanide	1% (w/v)
1,1-Diphenyl-2-picrylhydrazyl radical	60 μM
Acetone	
Titanium tetrachloride	10% (w/v)
Ammonium Hydroxide ($NH_3 \cdot H_2O$)	
30% Hydrogen peroxide	20 mM, 2mM
Riboflavin	1.5 μM

Supplementary Table S4. The List of primers used in this study.

EjSOD1	F: TCACTGGACCACACTCTATC
	R: CCAGCATTTCCTGTGGATT
EjSOD2	F: GACAAGCAGATTCCTCTCAC

	R: CATCCTTGCAGACCGATAAT
EjCAT1	F: CTGGGAGTTCCACAAGATTAC
	R: GCTCCTCCAACCTTAATAGC
EjCAT2	F: ACTCGACTCTTCGCCTATT
	R: AGTAGTCAACCTCCTCATCTC
EjAPX1	F: GCTGCTCAGATCTTCAATCA
	R: CGGAGCATAATCAGAGCATAG
EjAPX2	F: AGTACCCTGGTGTCTCATAC
	R: GACCTCAACTCTCCCATACT
EjACT (JN004223)	F: AATGGAACTGGAATGGTCAAGGC
	R: TGCCAGATCTTCTCCATGTCATCCCA
